# Diagnostic accuracy of de‐escalated surgical procedure in axilla for node‐positive breast cancer patients treated with neoadjuvant systemic therapy: A systematic review and meta‐analysis

**DOI:** 10.1002/cam4.4769

**Published:** 2022-05-03

**Authors:** Yu‐xin Song, Zheng Xu, Ming‐xing Liang, Zhen Liu, Jun‐chen Hou, Xiu Chen, Di Xu, Yin‐jiao Fei, Jin‐hai Tang

**Affiliations:** ^1^ The Department of General Surgery The First Affiliated Hospital of Nanjing Medical University Nanjing China

**Keywords:** breast cancer, neoadjuvant therapy, sentinel lymph node biopsy, targeted lymph node biopsy, targeted lymph node dissection

## Abstract

**Background:**

More initial clinical node‐positive breast cancer patients achieve axillary pathological complete response (ax‐pCR) after neoadjuvant systemic therapy (NST). Restaging axillary status and performing de‐escalated surgical procedures to replace routine axillary lymph nodes dissection (ALND) is urgently needed. Targeted axillary lymph node biopsy (TLNB) is a novel de‐escalated surgical strategy marking metastatic axillary nodes before NST and targeted dissection and biopsy intraoperatively to tailor individual axillary management.

**Methods:**

This study provided a systematic review and meta‐analysis to evaluate the feasibility and diagnosis accuracy of TLNB. Prospective and retrospective clinical trials on TLNB were searched from Pubmed, Embase, and Cochrane. Identification rate (IFR), false‐negative rate (FNR), negative predictive value (NPV), and rate of ax‐pCR were the outcomes of this meta‐analysis.

**Results:**

One thousand nine hundred and twenty patients attempted TLNB, with an overall IFR of 93.5% (95% confidence interval [CI] 90.1%–96.2%). IFR of three nodal marking methods, namely iodine seeds, clips, and carbon dye, was 95.6% (95% CI 91.2%–98.7%), 91.7% (95% CI 87.3%–95.4%), and 97.1% (95% CI 89.1%–100.0%), respectively. Of them, 847 patients received ALND, with an overall FNR of 5.5% (95% CI 3.3%–8.0%), and NPV ranged from 90.1% to 96.1%. Regression analysis showed that the overlap of targeted and sentinel biopsied nodes might associate with IFRs and FNRs.

**Conclusion:**

TLNB is a novel, less invasive surgical approach to distinguish initial node‐positive breast cancer that achieves negative axillary conversion after NST. It yields an excellent IFR with a low FNR and a high NPV. A combination of preoperative imaging, intraoperative TLNB with SLNB, and postoperative nodal radiotherapy might affect the future treatment paradigm of primary breast cancer with nodal metastases.

## Graphical abstract

Axillary surgeries for node‐positive neoadjuvant breast cancer could be de‐escalated. Targeted lymph node dissection has high feasibility and diagnosis accuracy. A stepwise treatment strategy combining imaging and radiotherapy is recommended.

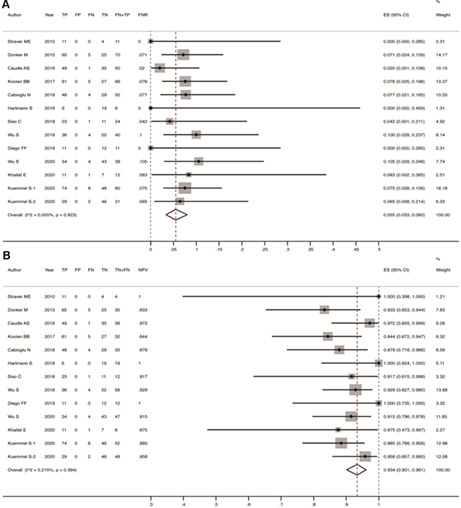

## 1 BACKGROUND

Axillary lymph node status is the most closely related factor affecting the survival and prognosis of breast cancer patients.^1^ With the increasing effectiveness of neoadjuvant systemic therapy (NST), nearly 30%–70% of locally advanced breast cancer achieved pathological complete response (pCR) in primary lesion and metastatic axillary lymph nodes, notably in the triple‐negative and human epidermal growth factor receptor 2 (Her‐2)‐amplified subtypes.^2^, ^3^ Nevertheless, to date, mandatory axillary lymph node dissection (ALND) was still the standard surgical approach for initial node‐positive (N+) patients after receiving NST endorsed by broad countries (Finland, Sweden, Romania) and international guidelines (Spanish Society of Medical Oncology, Germany S3 guideline).^4^, ^5^ Of whom, patients with complete eradication of the disease in axilla should be noticed, since they might be spared axillary clearance with a high risk of morbidity including lymphedema, numbness, paresthesia, and limited movement.^6^

Over the past decades, surgeons have been committed to exploring innovative approaches to precisely identify patients with axillary pCR (ax‐pCR). Sentinel lymph nodes biopsy (SLNB) gradually replaced ALND after NST in patients with clinically node‐negative (cN0) stage at diagnosis. The large‐scale, multi‐center clinical trials GANEA 2 and NSABP B‐27 proved that SLNB had a similar detection rate before and after NST, and the omission of ALND did not increase the nodal recurrence rate following observation of 47 months.^7^, ^8^ While regarding patients with initially axillary metastases, failure occurred when sentinel lymph nodes (SLNs) were reported as negative, but residual metastatic components were found in the other axillary nodes, with a widely varying incidence ranging from 8% to 40%.^9^, ^10^, ^11^ It might be a consequence of tumor‐induced lymphatic obstruction and treatment‐related fat necrosis and lymphatic return pathway changes.^12^ Though studies showed that the false‐negative incidence could be controlled to about 10% when using a dual‐tracer technique to remove three or more biopsied nodes, data from ACOSOG Z1071 and SENTINA reported that only 56% and 34% of patients met this requirement.^13^, ^14^ Thus, more targeted detection techniques were urgently needed for axillary conservation in the context of NST. Recently, American National Comprehensive Cancer Network recommended marking pathological‐confirmed metastatic lymph nodes before NST and intraoperatively dissecting the targeted lymph nodes (TLNs) and the dual‐tracer sentinel lymph nodes (SLNs) in initial N+ neoadjuvant breast cancer for axillary restaging (level of evidence and strength of recommendation, 2b), which was likewise supported by European Society for Medical Oncology, American Society of Breast Surgeons and Breast Committee of German Gynecological Oncology Group.^15^, ^16^ From the international conference, the Lucerne toolbox, 82% of panelists agreed selected node‐positive patients could forego ALND after NST.^17^ Feedback from the latest Saint Gallen panel showed that for initial positive lymph node status (pN1) with good clinical response, 86% of scholars considered targeted lymph nodes biopsy (TLNB) an optional solution to guide axillary management.^18^ Controversies arose when focusing on the standardized practice of TLNB, with 37% favoring avoiding ALND when one of one TLN was negative, 7% agreeing when two of two TLNs were negative, and 41% agreeing when three of three TLNs were negative. Additionally, there was no consensus on the selection criteria for the most suitable candidates before NST.

In our study, we defined TLNB as an intraoperative biopsy of one or two pathological‐confirmed metastatic lymph nodes marked prior to NST, while targeted axillary lymph node dissection (TAD) was the overall biopsy findings of TLNs, SLNs, and palpable axillary disease. This study assembled cutting‐edge information on the different nodal marking methods and targeted dissection techniques in node‐positive neoadjuvant breast cancer patients. We aimed to evaluate the diagnostic accuracy of TLNB for axillary restaging after NST and to explore whether the less invasive surgical strategy could replace ALND without compromising survival outcomes.

## 2 METHODS

### 2.1 Criteria for considering studies for this review

This article was based on the Preferred Reporting Items for Systematic Reviews and Meta‐Analyses (PRISMA) guidelines and AMSTAR (Assessing the methodological quality of systematic reviews) Guidelines.^19^ The study was registered on PROSPERO (https://www.crd.york.ac.uk/prospero/) with the registration number CRD42021238376. The first author (YS) systematically reviewed the randomized controlled trials, case–control studies, and cohort studies on marking pathological‐confirmed positive lymph nodes and targeted dissection among N+ neoadjuvant breast cancer. Reviews, case reports, and editorials were excluded.

Inclusion criteria and exclusion criteria were listed as follows: Inclusion criteria are as follows: (1) clinical node‐positive patients underwent core needle biopsy or fine‐needle aspiration before NST and were pathologically confirmed breast cancer and node metastasis; (2) patients were given priority to chemotherapy, targeted therapy, or endocrine therapy without axillary surgery; (3) patients who marked the metastatic nodes and received TLNB in operation. Exclusion criteria are as follows: (1) no pathologic evidence of axillary lymph node metastasis was found before NST; (2) patients with distant metastasis or who have received prior ipsilateral axillary treatments (surgery, radiotherapy).

The primary outcomes of this study were the identification rate (IFR), false‐negative rate (FNR), and negative predictive value (NPV). The second outcome was the ax‐pCR rate. Successful identification of TLNs was removing axillary lymph nodes containing clips, iodine seeds, or carbon dye confirmed by specimen radiography or visualization of pathology during the TLNB. Eligible patients were enrolled and analyzed the IFR. Patients with successful TLNB and routine ALND were assigned to a separate group to calculate the FNR, NPV, and ax‐pCR rate. Pathology of ALND was regarded as the gold standard of axillary staging. Data of studies were collated into a 2*2 contingency table consisting of the numbers of true positive (TP), false negative (FN), false positive (FP), and true negative (TN). The TN and TP were the patients whose pathological findings of the TLNB (presence or absence of residual tumor) were consistent with that of the ALND. The FN was the patients without evidence of tumor residual in TLNs but with tumorous components in other axillary lymph nodes (ALNs). The FP was the patients of whom tumor residuals were reported only in TLNs but not in other ALNs. Cause we considered the TLNs were part of ALNs, when FP was reported more than 0, we would adjust and add the number to TP before the quantitative analysis. FNR was the probability of all metastatic patients being misclassified as patients without tumor residual in axilla according to TLNB, calculated as FN/(FN + TP). NPV was the probability of all the negative patients being diagnosed accurately based on TLNB, calculated as TN/(FN + TN). Ax‐pCR rate was calculated by TN/sample size. Considering the TPs might also be a population that could avoid ALND after TAD, the false‐positive rate (FPR) and positive predictive value (PPV) were analyzed before putting statistical FP at 0.

### 2.2 Search methods for identification of studies

Two authors (YS and ZX) independently searched for articles from Pubmed, Embase, and Cochrane collaboration up to February 1, 2021. Subject word and random word were used in document retrieval, and details are in Appendix S1.

### 2.3 Study collection and quality assessment

Duplications were automatically identified and deleted through Endnote. Two authors (YS and ZL), respectively, browsed the titles and abstracts of articles and screened out qualified documents. Quality Assessment of Diagnostic Accuracy Studies 2 (QUADAS‐2) was used to evaluate the literature quality. In case of disagreements, another author (JT) participated and made a judgment.

### 2.4 Data extraction

Two authors (YS and ZX) extracted data independently. The following items were extracted: basic information about clinical trials (author, year of publication, study type, sample size), clinical characteristics of subjects (proportion of Her‐2 positive and triple‐negative subtypes, aspiration methods, node status before and after NST, NST regimens), details of de‐escalated surgical procedure (marking criteria, marking method, localization method, successful rate, confirming removal of marked nodes, and addition of SLNB), qualities of SLNB (failure rate, median number of SLNs, the overlap rate of TLNs and SLNs), application of immunochemistry (IHC), IFR, diagnosis accuracy of de‐escalated surgical procedure (TP, FP, FN, TN). Even exacted from the same study, the group could be more than one if the biopsied and localized methods differed if the outcomes could be traced to the corresponding population.

### 2.5 Statistical analysis

Data synthesis was based on STATA/SE Statistical Software, version 15.0 (StataCorp LP). FNR, NPV, and IFR were calculated using random‐effects models and fix‐effects models with the help of metaprop command.^20^ The test results were presented in forest plots, including pooled estimates and 95% confidence intervals (95% CIs). *χ* ^2^ test and *I* ^2^‐index were used to assess the heterogeneity between each group. *p* value ≤0.05 (bilateral) indicated a statistically significant difference. Sensitivity analysis, subgroup analysis, and meta‐regression analysis were performed to assess the sources of heterogeneity. We considered factors such as sample sizes, marking methods, the addition of SLNB, localization managements (whether to accept imaging‐assisted positioning of labeled lymph nodes and the precise localization method), and node stage after NST. Depending on the Cochrane Handbook for Diagnostic Test Accuracy Reviews, publication bias was not assessed.^21^ To explore the relationship between the overlap rate of biopsied nodes (SLNs and TLNs) and primary outcomes, a scatter chart with linear regression analysis was plotted using GraphPad Prism 8 Version 8.2.1.

## 3 RESULTS

### 3.1 Study selection

**FIGURE 1:**
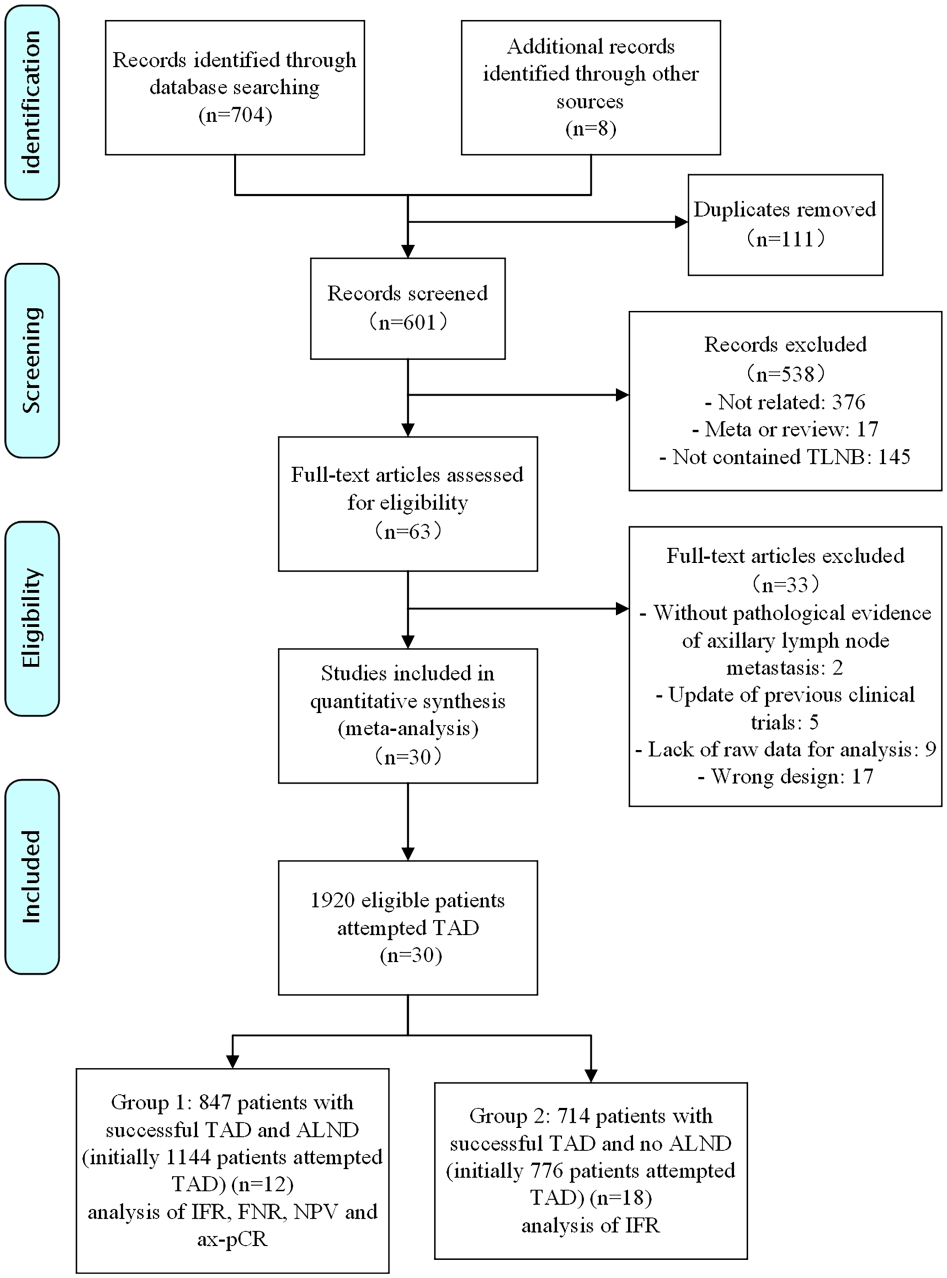
Flow diagram for literature screening

A total of 712 articles were identified. Of the duplication, 601 articles were screened for titles and abstracts. Finally, 30 articles documented the details of eligible patients ready to undergo TLNB. The flow diagram for literature screening is listed in Figure 1.

### 3.2 Study characteristics

Briefly, a sum of 1920 patients was enrolled, of whom 847 patients with ALND after successfully retrieving TLNs were assigned to Group 1, 714 patients without final pathological examination of axilla were assigned to Group 2. Three marking methods were recorded: radioactive seed (iodine‐125), clip, and carbon dye. General characteristics are presented in Table 1 and details in Table 2 (Group 1) and Table 3 (Group 2), respectively.

**TABLE 1:** General characteristics of the included studies

Author	Year of publication	Country	Study type	Sample size^a^	Ax‐pCR rate, %	Aspiration methods	N‐Stage before NST, %	No. of abnormal nodes on ultrasound, %	Proportion of Her‐2 positive and TNBC, %	NST regimens for Her‐2 negative	NST regimens for Her‐2 positive
Groups with ALND											
Straver^34^	2010	Netherland	S, P	15	26.7	FNA	cN1: 100	1: 100	60 (26.7, 33.3)	EC/EC → DX	PacCb + Tra
Donker^35^	2015	Netherland	S, P	100	26.3	FNA	cN1: 60.2; cN2: 12.6; cN3: 27.2	/	47.6 (26.2, 21.4)	EC/DX	PacCb + Tra
Caudle^59^	2016	USA	S, R	90	41.2	FNA	/	1: 38.5; 2: 13.5; 3: 21.9; 4: 26.0	39.6 (18.8, 20.8)	E ± T based chemotherapy/endocrine therapy	Addition of anti‐Her‐2
Koolen^36^	2017	Netherland	S, P	125	29	FNA	cN1: 63.4; cN2–3: 36.6	/	51.6 (30.1, 21.5)	EC/EC → DX	PacCb + Tra
Cabioglu^53^	2018	Turkey	S, P	99	35.8	FNA	cN1: 76.7; cN2: 18.6; cN3: 4.7	/	19.8 (7.0, 12.8)	EC → T	EC → T + Tra
Hartmann^24^	2018	Germany	S, P	24	76	CNB	cN1: 83.3; cN2: 10.0; cN3: 6.7	/	73.3 (36.7, 36.7)	E ± T ± Pac based chemotherapy	Addition of anti‐Her‐2
Siso^37^	2018	Spain	S, P	46	31.4	FNA	/	1: 50.0; 2: 19.6; 3: 19.6; >3: 10.9	32.6 (23.9, 8.7)	EC → T/aromatase inhibitors	Addition of anti‐Her‐2 (Tra ± Per)
Wu^26^	2018	China	S, P	95	59.8	FNA	cN1: 85.9; cN2: 4.1	/	57.6 (Her‐2, TNBC NR)	ET/CbPac/D	Addition of Tra (±Per)
Diego^42^	2019	Spain	S, P	23	52.2	FNA	cN1: 47.8; cN2: 52.2	/	69.6 (52.2, 17.4)	EC → D/PacCb→EC	DCb + Per + Tra
Wu^25^	2020	China	S, P	84	53.1	FNA/CNB	cN1: 78.2; cN2: 20.3; cN3 1.5	/	71.4 (47.4. 24.1)	ET/PacCb/D	Addition of Tra (±Per)
Khallaf^44^	2020	Egypt	S, P	20	40	FNA	cN1: 85.0; cN2: 15.0	/	45.0 (35.0, 10.0)	E ± T based chemotherapy	Addition of Tra
Kuemmel‐1^22^	2020	Germany	M, P	194	48.4	FNA/CNB	/	1: 49.3; 2: 19.7; ≥3: 31.0	54.8 (41.3, 13.5)	Chemotherapy/endocrine therapy	Addition of anti‐Her‐2
Kuemmel‐2^22^				229	40.30			1: 59.7; 2: 18.2; ≥3: 22.1	44.2 (24.7, 19.5)		
Groups without ALND											
Choy^43^	2015	USA	S, P	9	NR	FNA/CNB	cN0: 11.1; cN1: 77.8; cN2: 11.1	/	41.7 (16.7, 25.0)	Chemotherapy	Addition of anti‐Her‐2
Plecha^66^	2015	USA	S, R	91	NR	CNB	NR	NR	NR	Chemotherapy	Addition of anti‐Her‐2
Diego^38^	2016	USA	S, R	30	63.3	NA	NR	NR	93.3 (53.3, 40.0)	Chemotherapy	Addition of anti‐Her‐2
Nguyen^67^	2017	USA	S, P	34	NR	FNA	NR	NR	NR	Chemotherapy	Addition of anti‐Her‐2
Kim^54^	2018	Korea	S, P	11 (13 clips)	36.4	FNA/CNB	/	1: 10; 2: 40.0; 3: 50.0	70.0 (55.0, 15.0)	Chemotherapy	Addition of anti‐Her‐2
Park^46^	2018	Korea	S, P	20	50	FNA	cN1: 60.0; cN2: 25.0; cN3: 15.0	/	45.0 (25.0, 20.0)	EC → T/P	EC → T/P + Tra
Allweis^68^	2019	Israel	S, R	63	NR	FNA/CNB	NR	NR	50.8 (33.3, 17.5)	Chemotherapy/endocrine therapy	Addition of anti‐Her‐2
Hellingman^39^	2019	Netherland	M, R	37 (38 axillae)	27	FNA	cN1: 60.5; cN2: 23.7; cN3: 15.8	/	26.3 (10.5, 15.8)	EC/EC → Pac/EC → CbPac/CbPac/endocrine therapy	CbPac + Tra + Per
Kim^45^	2019	Korea	S, P	28	46.4	FNA	cN1: 67.9; cN2: 17.9; cN3: 14.3	/	39.3 (28.6, 10.7)	EC → T	EC → T + Tra
Patel^69^	2019	USA	S, P	37	62.1	FNA/CNB	cN0: 12.8; cN1: 68.2; cN2: 14.9; cN3: 4.3	/	70.2 (40.4, 29.8)	Chemotherapy	Addition of anti‐Her‐2
Simons^70^	2019	Netherland	M, R	139	NR	FNA/CNB	cN1: 73.4; cN2: 18.7; cN3: 7.9	/	51.1 (33.1, 18.0)	Chemotherapy	Addition of anti‐Her‐2
Balasubramanian^71^	2020	UK	S, R	25	36	CNB	NR	NR	NR	Chemotherapy	Addition of Tra + Per
Goyal^48^	2020	UK	M, P	22	NR	FNA/CNB	NR	NR	Her‐2 38.1	Chemotherapy	NR
Lim^61^	2020	Korea	S, P	14 (20 clips)	45	FNA	/	1: 50.0; 2: 21.4; 3: 28.6	NR	E + T based chemotherapy/immunotherapy	Addition of anti‐Her‐2
Laws^51^	2020	USA	S, R	57	48.2	FNA/CNB	cN1: 94.7; cN2: 5.3	/	71.9 (45.6, 26.3)	EC → T/EC → TCb/TAC/endocrine therapy	TC + Tra + Per/EC → T + Tra + Per/T + Tra + Per/T + Tra
Spautz^47^	2020	Brazil	M, P	74	NR	FNA	cN1: 85.3; cN2: 14.7	/	52.0 (31.7, 20.3)	E + T based chemotherapy	Addition of anti‐Her‐2
Kanesalingam^23^	2020	Australia	S, P	37	NR	FNA/CNB	NR	NR	40.5 (32.4, 8.1)	Chemotherapy	Addition of anti‐Her‐2
Beniey^72^	2020	Canada	S, R	39	50	NR	NR	NR	NR	Chemotherapy	Addition of anti‐Her‐2

Abbreviations: ALND, axillary lymph node dissection; Ax‐pCR, axillary pathological complete response; C, cyclophosphamide; Cb, carboplatin; cN, clinical node stage (TMN stage); CNB, core needle biopsy; D, docetaxel; E, doxorubicin; FNA, fine‐needle aspiration; Her‐2, human epidermal growth factor receptor 2; M, multicenter; NR, not reported; NST, neoadjuvant systemic therapy; P, prospective; Pac, paclitaxel; Per, pertuzumab; R, retrospective; S, single center; T, taxane; TNBC, triple‐negative breast cancer; Tra, trastuzumab; X, capecitabine.

^a^ The sample size was the number of patients who attempted TLNB.

**TABLE 2:** Clinical characteristics of downstage axillary managements in Group 1

Author	Sample size^a^	Initial cN1 rate, %	N‐Stage after NST	Biopsy methods	TLNB						SLNB			TLNs were SLNs, %	IHC
					Marking before NST	Screening criteria for TLNs	Localization of TLNs before surgery	Successful localization rate, %	Confirming removal of TLNs	IFR, %	SLNB method, %	SLNs not identified, %	Median number of SLNs		
Straver 2010^34^	15	100	/	TLNB	Iodine‐125 seed (STM1251)	The largest metastatic ALN	Gamma probe	100	Detecting the iodine‐125 source	100	/	/	/	/	Yes, only when HE (−)
Donker 2015^35^	95	60.2	/	TLNB	Iodine‐125 seed (STM1251)	The previous proven metastatic ALN	Gamma probe	100	/	97	/	/	/	/	Yes, only when HE (−)
Caudle 2016^59^	85	74	/	TLNB + SLNB	Clip (HydroMark)	The most suspicious ALN	Wire localization/iodine‐125 seed localization + gamma probe	100	Specimen radiograph	94.4	Single (blue or 99mTc) 44.9 dual 55.1	6	NR	75.4	Yes, if TLN is SLN
Koolen 2017^36^	93	63.4	/	TLNB	Iodine‐125 seed	The largest metastatic ALN	Gamma probe	100	/	97.6	/	/	/	/	Yes, only when HE (−)
Cabioglu 2018^53^	81	76.7	cN0	TLNB + SLNB	Clip (766914100SST V Mark BreastBiopsy Site Marker)	The most suspicious ALN	/	/	Specimen radiograph	97	Blue 57.0 dual 43.0	12.1	2	81.4	Yes, if TLN is SLN
Hartmann 2018^24^	25	83.3	/	TLNB + SLNB	Clip (HydroMark)	The largest metastatic ALN	Wire localization	96	Specimen radiograph	70.8	Dual	13	1.6	35.7	Yes, not routinely
Siso 2018^37^	35	89.1	/	TLNB + SLNB	Clip (HydroMark)	The previous proven metastatic ALN	High frequency probe + gamma probe	100	/	95.7	Dual	0	3	77.1	Yes
Wu 2018^26^	92	85.9	/	TLNB + SLNB	Clip	The previous proven metastatic ALN	Wire localization	95	Specimen radiograph	96.8	Blue ± 99mTc	NR	NR	66.7	No, ITPC
Diego 2019^42^	23	47.8	/	TLNB + SLNB	Clip (HydroMark, Tumark)	The most suspicious ALN	Wire localization	91	/	95.7	99mTc	17.4	NR	60.9	NR
Wu 2020^25^	81	78.2	/	TLNB + SLNB	Clip	The previous proven metastatic ALN	Wire localization	NR	Specimen radiograph	97.6	Dual	NR	NR	NR	NR
Khallaf 2020^44^	19	85	cN0	TLNB + SLNB	Carbon (Spot)	The most suspicious ALN	/	/	/	95	Blue	NR	NR	87.5	NR
Kuemmel‐1 2020^22^	126	/	/	TLNB	Clip	The most suspicious ALNs (one or two)	Wire localization/palpation /stereotactic localization	NR	Specimen radiograph	67	/	/	/	/	Yes, not routinely
Kuemmel‐2 2020^22^	77	/		TLNB + SLNB						86.9	99mTc ± blue	15.4	1	63.6	

Abbreviations: ALN, axillary lymph node; ALND, axillary lymph node dissection; IFR, identification rate; IHC, immunohistochemistry; NR, not reported; NST, neoadjuvant systemic therapy; SLNB, sentinel lymph node biopsy; TLN, targeted lymph node; TLNB, targeted lymph node biopsy.

^a^ The sample size was the number of patients had successful TLNB.

**TABLE 3:** Clinical characteristics of downstage axillary managements in Group 2

Author	Sample size^a^	Initial cN1 rate, %	N‐Stage after NST	ALND rate, %	Marking methods	TLNB						SLNB			TLNs were SLNs, %	IHC
						Marking before NST	Screening criteria for TLNs	Localization of TLNs	Successful localization rate, %	Confirming removal of TLNs	IFR, %	SLNB method, %	SLNs not identified, %	Median number of SLNs		
Choy 2015^43^	9	88.9	/	44.4	TLNB + SLNB	Carbon (Spot)	The previous proven metastatic ALN	/	/	/	100	99mTc ± blue	0	2.7	100	NR
Plecha D‐1 2015^66^	67	/	/	71.4	TLNB + SLNB	Clip (HydroMARK)	The previous proven metastatic ALN	Wire localization	100	Specimen radiograph	97	99mTc ± blue	NR	NR	NR	NR
Plecha D‐2 2015^66^	24							/	/		83.3					
Diego 2016^38^	30	/	cN0	83.3	TLNB + SLNB	Clip	The most suspicious ALN	Iodine‐125 seed localization + gamma probe	96.7	Specimen radiograph	96.7	Dual	NR	NR	66.7	Yes, not routinely
Nguyen TT‐1 2017^67^	20	/	/	76.5	TLNB + SLNB	Clip	The most suspicious ALN	Iodine‐125 seed localization + gamma probe	80	Specimen radiograph	100	Blue ± 99mTc	NR	NR	NR	NR
Nguyen TT‐2 2017^67^	14							/	/		78.6					
Kim 2018^54^	11 (13 clips)	/	/	54.5	TLNB + SLNB	Clip (LigaClip MCA MSM20)	The most suspicious ALNs (one or two)	Wire localization	100	Palpation/specimen radiograph	92.3	Dual	NR	2.2	70.8	NR
Park 2018^46^	20	60	/	60	TLNB + SLNB	Carbon (Charcotrace)	The largest metastatic ALN	/	/	/	100	Blue ± 99mTc	0	3	75	Yes, not routinely
Allweis 2019^68^	63	/	/	NR	TLNB + SLNB	Carbon (Spot)	The most suspicious ALNs (one or two)	/	/	/	95.2	99mTc	8.9	NR	80	NR
Hellingman 2019^39^	37 (38 axillae)	60.5	/	26.3	TLNB	Clip	The largest metastatic ALN	99mTc injection + gamma probe	97.4	Specimen radiograph	86.8	/	/	/	/	Yes
Kim 2019^45^	28	67.9	/	NR	TLNB + SLNB	Clip (ULTRACLIP)	The most suspicious ALN	Carbon tatoo	100	/	96.4	Dual	14.3	NR	62.5	NR
Patel 2019^69^	37	68.1	/	32.4	TLNB + SLNB	Carbon (Spot)	The most suspicious ALNs (one or two)	/	/	/	100	Blue ± 99mTc	0	3.7	100	NR
Simons‐1 2019^70^	68	73.4	/	22.3	TLNB + SLNB	Iodine‐125 seed	The most suspicious ALN	/	/		/	Dual 54.7 blue 5.8 99mTc 39.6	dual 14.5 single 9.5	NR	64.6	Yes, not routinely
Simons‐2 2019^70^	59					Clip		Wire localization	98.3							
Simons‐3 2019^70^	12							seed localization + gamma probe	100							
Balasubramanian 2020^71^	25	NR	/	NR	TLNB + SLNB	Clip (HydroMark)	The largest metastaticALN	Wire localization	100	Specimen radiograph	92	Dual	0	NR	87	Yes, not routinely
Goyal 2020^48^	22	NR	/	81.9	TLNB	Carbon (Spot or Black Eye)	The most suspicious ALN	/	/	/	63.6	/	/	/	100*	Yes, only isolated tumor cells or micrometastases
Lim 2020^51^	14 (20 clips)	100	/	NR	TLNB + SLNB	Clip (UltraCor Twirl, HydroMARK, UltraClip Dual Trigger or UltraClip markers)	The previous proven metastatic ALNs (one or two)	Skin marking + needle localization	100	Specimen radiograph	90	/	/	/	/	NR
Laws 2020^56^	57	94.7	/	38.6	TLNB + SLNB	Clip (HydroMARK)	the most suspicious ALN	Special tag localization	93	Specimen radiograph	89.5	Dual	NR	NR	NR	NR
Spautz 2020^47^	74	85.3	/	NR	TLNB + SLNB	Carbon (4% CMS)	The most suspicious ALN	/	/	/	100	Blue	12.2	2.3	62.2	NR
Kanesalingam‐1 2020^23^	12	/	cN0	35.1	TLNB + SLNB	Clip	The most suspicious ALN	Wire localization/iodine‐125 seed localization + gamma probe	100	Specimen radiograph	100	Dual	NR	NR	86.2	NR
Kanesalingam‐2 2020^23^	25							/	/		68					
Beniey 2020^72^	39	/	cN0	50	TLNB + SLNB	Iodine‐125 seed	The previous proven metastatic ALN	Gamma probe	100	Specimen radiograph	87.2	NR	NR	NR	NR	NR

Abbreviations: ALN, axillary lymph node; ALND, axillary lymph node dissection; IFR, identification rate; IHC, immunohistochemistry; NR, not reported; NST, neoadjuvant systemic therapy; SLNB, sentinel lymph node biopsy; TLN, targeted lymph node; TLNB, targeted lymph node biopsy.

^a^ The sample size was the number of patients who attempted TLNB.

### 3.3 Risk of bias and applicability

**FIGURE 2:**
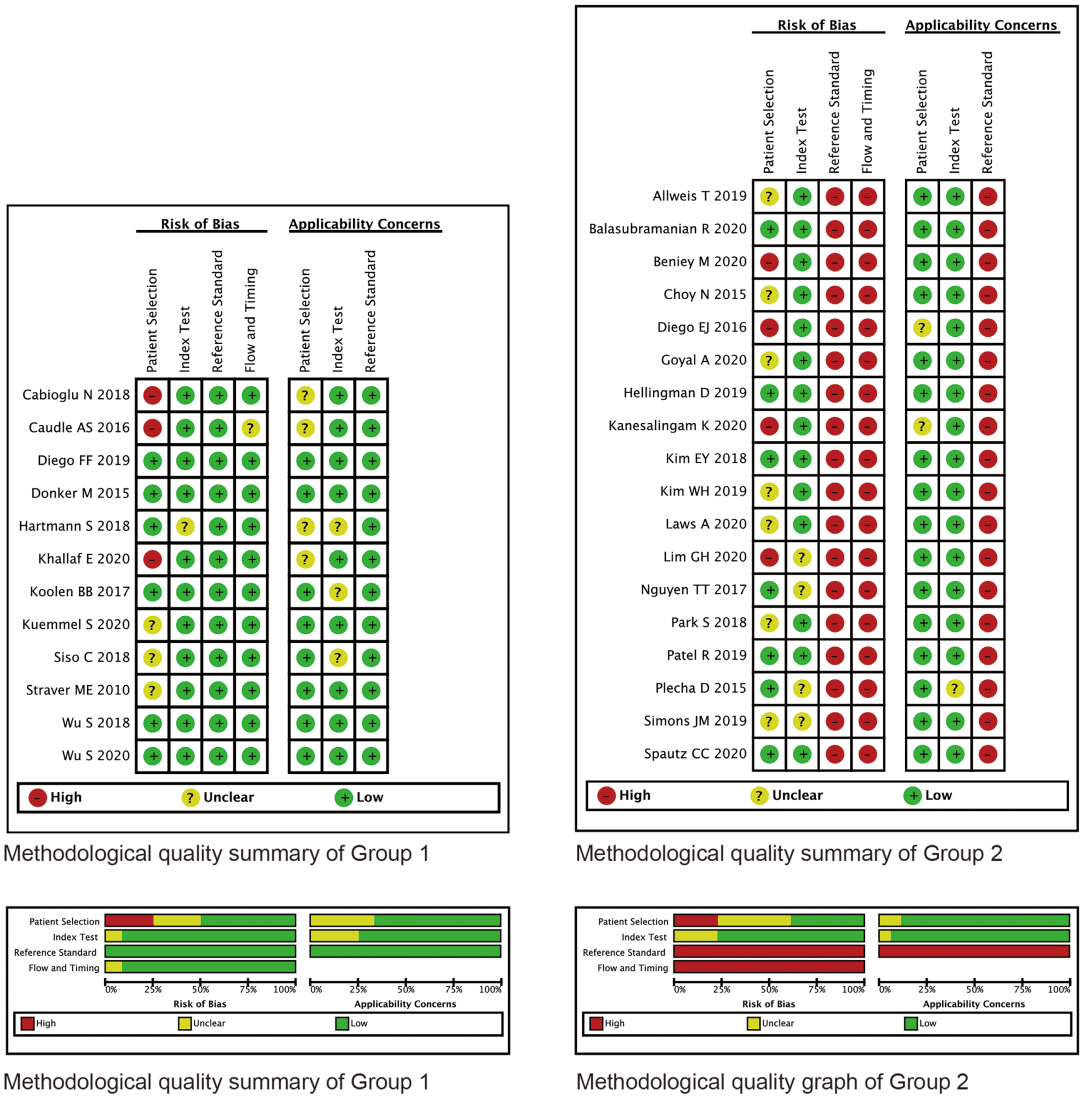
Methodological quality summaries and graphs of included studies

Methodological quality assessments are shown in Figure 2. Overall, Group 1 had a low risk of bias. Two trials with a high risk of bias only included patients with clinically lymph nodes complete response (cN0) after NST, which was not consecutive over the time range of patient selection. Risks of bias for reference standard and flow and timing were rated high in all the 18 trials in Group 2 because the reference standard (ALND) was not performed as part of the trial protocol.

### 3.4 Results of individual studies

#### 3.4.1 IFR

The pooled IFR was 93.5% (95% CI 90.1%–96.2%, *I* ^*2*^ = 81.383%, *p* = 0.000) of 1920 patients who attempted axillary node marking, suggesting a considerable heterogeneity between groups. A random‐effect model was used for subsequent analysis.

Sensitivity analysis identified that the *I* ^*2*^‐statistic decreased to 56.448% after removing 2 studies (Kanesalingam‐2, 2020 and Kuemmel, 2020).^22^, ^23^ In the former trial, the extremely low IFR of 69% was significantly associated with the lack of preoperative wire localization, compared to a 100% detection rate for the successful positioning group in the same study. The latter was a large prospective clinical trial involving more than 50 canters, whose investigators noted that IFR tended to be lower in centers with <20 TLNB cases, suggesting differences in operator mastery of the techniques between centers might lead to deviations. The adjusted pooled IFR was 95.2% (95% CI 93.0%–97.0%, *p* = 0.000).

**FIGURE 3:**
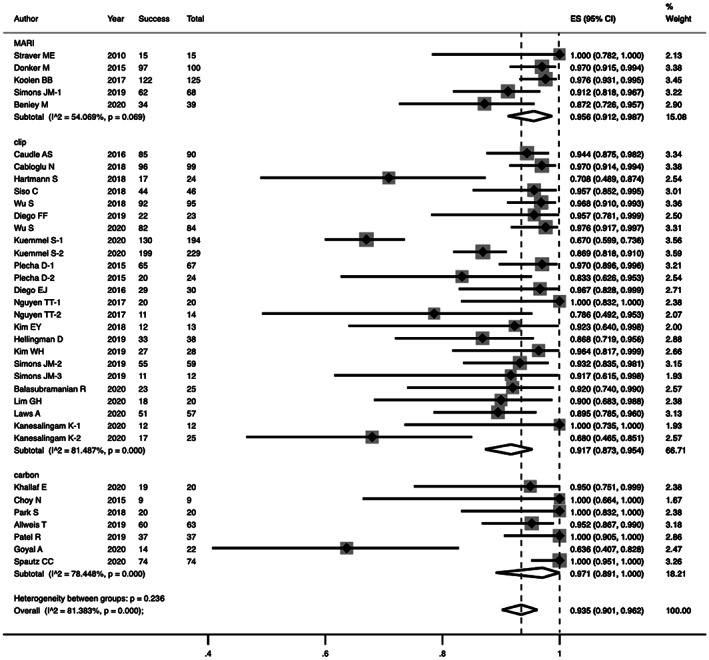
Forest plot of the identification rate of TLNB, grouped by marking method (MARI, clip or carbon)

Subgroup analysis showed, the pooled IFR of iodine seed (347 cases), clip (1328 cases) and carbon dye (245 cases) were 95.6% (95% CI 91.2%–98.7%), 91.7% (95% CI 87.13%–95.4%), and 97.1% (95% CI 89.1%–100.0%), respectively (heterogeneity between subgroups: *p* = 0.236) (Figure 3). Based on localization methods, the pooled IFR was 96.1% (95% CI 92.3%–98.9%) in groups using γ probe and 94.5% (95% CI 89.9%–97.9%) in groups using wire, compared to 93.3% (86.3%–98.2%) in groups without presurgical positioning. One concern was that the excellent IFR of the carbon‐labeled group (which cannot be localized under imaging assistance) raised the overall level of the unlocalized population. When we dropped the seven studies with carbon dye, the IFR of the unlocalized and the localized groups was 86.8% (95% CI 74.1%–95.9%) and 93.5% (95% CI 89.5%–96.7%), respectively.

Meta‐regression analysis revealed the potential correction between IFR with marking methods (*p* = 0.077) and sample sizes (*p* = 0.021), but not with node stage after NST, combination with SLNB, and localization management. Interestingly, the linear regression analysis found that groups with high overlap between SLNs and TLNs tended to have a better lymph node detection rate (Figure 5A).

#### 3.4.2 FNR and NPV

**FIGURE 4:**
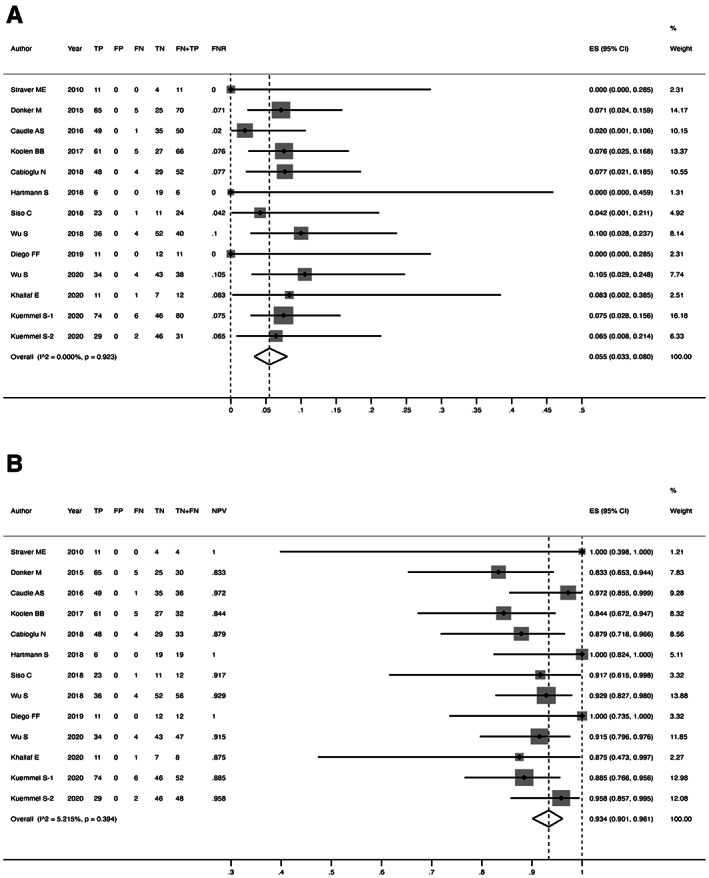
(A) Forest plot of the FNR of TAD; (B) Forest plot of the NPV of TAD

Figure 4 showed forest plots for the FNR and NPV in all 847 patients receiving ALND. The overall FNR was 5.5% (95% CI 3.3%–8.0%), with *I* ^*2*^ statistic of 0% (*p* = 0.923). The pooled NPV ranged from 90.1% to 96.1% (*I* ^*2*^ = 5.215%, *p* = 0.394). Heterogeneity between groups was slight. Thus, a fixed‐effect model was used for subsequent analysis.

Methods for labeling nodes were documented: radioactive seeds in 203 patients, clips in 625 patients, and carbon dye in 19 patients. FNR for each method was 5.8% (95% CI 2.1%–10.7%), 5.4% (95% CI 2.9–8.5%) and 8.3% (95% CI 0.2%–38.5%), with NPV of 87.0% (95% CI 76.5%–95.2%), 94.1% (95% CI 90.9%–96.7%) and 87.5% (95% CI 47.3%–99.7%), respectively. Four studies implemented TLNB alone and nine studies simultaneously combined SLNB as intraoperative biopsies, with a pooled FNR of 5.1% (95% CI 2.3%–8.6%) and 6.3% (95% CI 3.2%–10.1%). The difference between the two groups was not statistically significant (*p* = 0.878). Strangely, three of nine combining biopsied studies adopted dual‐tracer for SLNB, presented an overall FNR of 5.6% (95% CI 0.6%–13.7%), compared to two studies that used single‐tracer of 2.9% (95% CI 0.0%–16.3%) (the remaining 4 studies mixed both methods). The addition of SLNB likewise improved the efficiency of detecting negative patients, with a higher NPV of 95.1% (95% CI 91.7%–97.8%) in the combined group and 88.3% in the single group (heterogeneity between groups: *p* = 0.024). After NST, 100 patients with excellent therapeutic effect converted to cN0, with a pooled FNR of 7.0% (95% CI 1.4%–15.4%), of whom the probability of misdiagnosed positive cases as FNs was not decreased. Totally 747 patients received localization of TLNs, among whom 203 received imaging‐guide wire localization preoperatively, and 221 received γ‐probe guide I‐125 setting intraoperatively. The FNR was 6.4% (95% CI 1.5%–13.3%) and 5.8% (95% CI 2.1%–10.7%) for the former two groups, compared to 7.0% (95% CI 1.4%–15.4%) for the remaining patients without localization.

**FIGURE 5:**
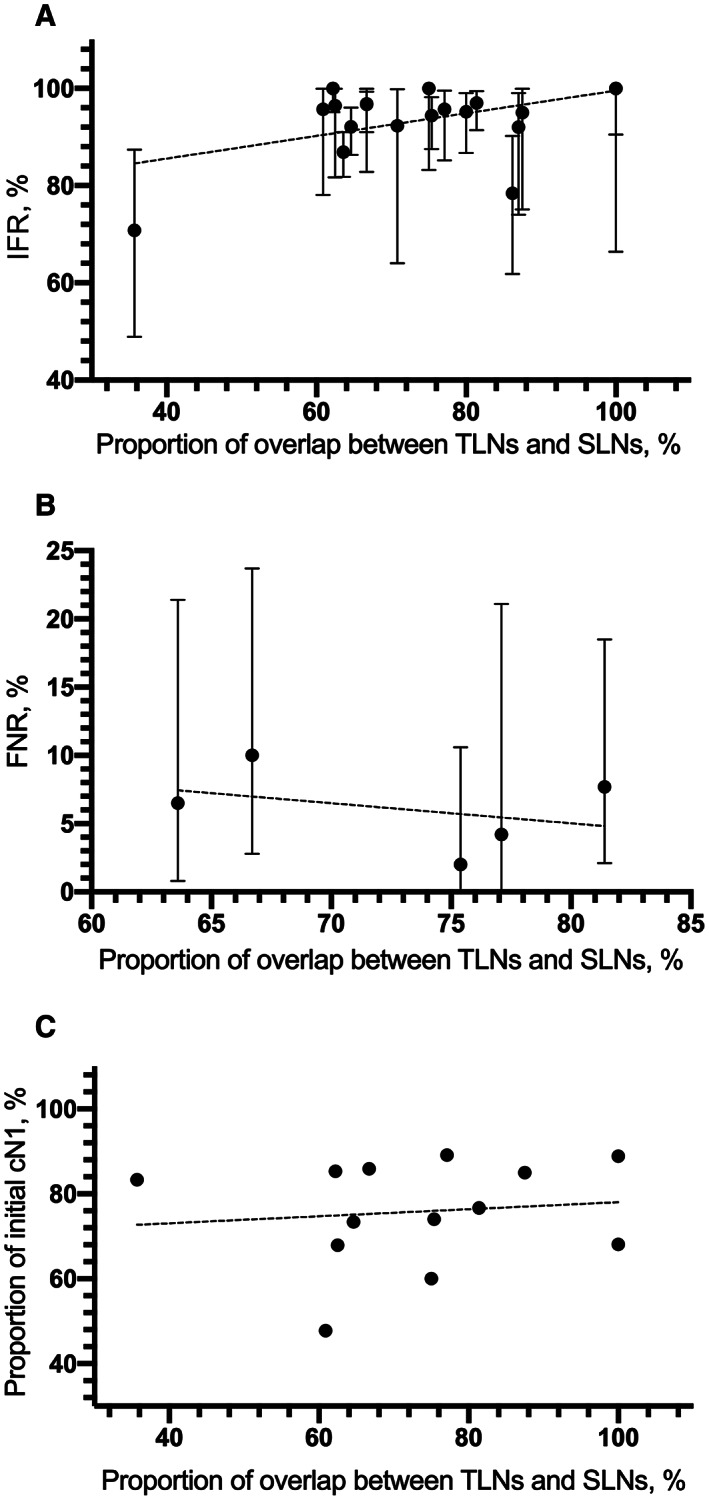
Linear regression analysis between overlap rates of TLNs and SLNs and (A) IFNs; (B) FNRs; (C) initial cN1 rate

Meta‐regression analysis confirmed that higher NPV was associated with the combination of SLNB (*p* = 0.024) and different marking methods (*p* = 0.039). Besides this, regression analysis did not detect the correlation between the factors we mentioned in the Method with FNR and NPV. Additionally, linear regression analysis showed a slight association between the proportion of overlap between SLNs and TLNs and FNR (excluding 3 studies with less than 30 patients) (Figure 5B) and the initial cN1 rate (Figure 5C).

#### 3.4.3 Ax‐pCR rate

The overall ax‐pCR rate in group 1 was 42.9% (95% CI 35.2%–50.7%) in a random‐effect model, with the *I* ^*2*^‐statistic of 79.017% (*p* = 0.000), suggested significant heterogeneity between groups.

Sensitivity analysis showed *I* ^*2*^ statistic decreased to 15.805% (*p* = 0.302) after removal 4 studies (Kuemmel‐2, 2020; Hartmann, 2018; Wu, 2018; Wu 2020).^22^, ^24^, ^25^, ^26^ The commonality of the 4 studies was high ax‐pCR rates, and more than 50% of cases belonged to Her‐2‐positive or triple‐negative subtypes. The corrected pooled ax‐pCR rate was 34.1% (95% CI 29.7%–38.6%) after removing the 4 studies.

Subgroup analysis demonstrated that the pooled ax‐pCR rate was 46.8% in groups with more than half of Her‐2‐positive and triple‐negative, compared to 38.6% in groups of that less than 50% (Figure S1). Strangely, we found a statistical difference in ax‐pCR rate between groups marking with radioactive seeds (27.3%, 95% CI 21.2%–33.8%) and groups marking with clips (48.3%, 95% CI 40.0%–56.6%) (*p* = 0.000). Ax‐pCR rate tended to be higher in the group with high initial cN1 proportion (cut‐off value: 75%, 48.1% vs. 34.9%, *p* = 0.035).

In a multiple‐covariates contained meta‐regression model, results showed an association with marking methods (*p* = 0.037). Furthermore, the chemotherapy protocols, application of targeted medicine, and initial tumor burden might also contribute to different therapeutic effects between groups.

#### 3.4.4 FPR and PPV

The overall FPR of Group 1 was 6.3% (95% CI 0.1%–17.5%), and PPV ranged from 84.7% to 99.8% (Figure S2), which indicated an excellent diagnostic in patients with tumor residue after NST.

## 4 DISCUSSION

Axillary management for primary advanced breast cancer has tended to be de‐escalated and individualized with the increasingly improved and targeted treatment tools. SLNB after NST had been widely incorporated in clinical practice for years. Previous retrospective series reported by Geng et al. showed a diagnostic accuracy of SLNB in initial cN0 breast cancer after NST was 99%, with a favorable FNR of 6%.^27^ However, when we turn our attention to initial pathological‐confirmed N+ breast cancer receiving SLNB, a meta‐analysis from Tee et al. depicted an overall IFR of 90% and an unacceptable pooled FNR up to 14%.^28^ The test efficiency tended to be higher in the population with axillary status converted to cN0 after NST. Several strategies were emerged to decrease the FNR of SLNB after NST, including the addition of dual‐tracers with more than three nodes biopsied, innovative tracing methods (indocyanine green fluorescence dye), intraoperative contrast‐enhanced ultrasonography, and lymphoscintigraphy.^27^, ^29^, ^30^, ^31^, ^32^ The next question was the therapeutic safety after exempting ALND based on biopsy results of SLNB. Data from the Mayo Clinic revealed that of 602 cases, the 2‐year freedom from regional recurrence was 99.1% in patients undergoing SLNB compared to 96.4% for those undergoing ALND after NST. Coincidentally, a recent retrospective mono‐institutional study on the survival benefits of standard SLNB after NST reported a superior 5‐ and 10‐year overall survival of 89.8% and 80.1% in initial cN1/2 patients who became cN0 after NST, and 92.0% and 81.5% in initial cN0 patients.^33^ The above evidence considered SLNB a feasible means of managing de‐escalated surgical procedures following NST in the context of surgeon adherence to certain technical parameters. Strategies with more targeted biopsy procedures, lower morbidity, and stricter stratification criteria were progressively supported.

To our knowledge, this is the first meta‐analysis comprehensively summarized clinical trials of premarking lymph node and targeted dissection technique, which is a turning point in surgical oncology to help identify downstage breast cancer with initial node metastasis. To date, several nodal marking methods were developed, usually based on techniques already utilized to localize breast lesions. Marking axillary nodes with radioactive iodine‐125 seeds (MARI) under ultrasound guidance was first attempted by Straver et al. in 2010, reaching a diagnostic accuracy of 100%.^34^ MARI presented favorable node detection rates with intraoperative γ probe detectors.^34^, ^35^, ^36^, ^37^, ^38^, ^39^ Experience showed that the placement of iodine seeds did not distract the recognition of the SLNs traced by 99mTc.^40^, ^41^ Despite the low dose of radioactivity, this method required complete radiation safety supervision throughout the process and therefore cannot be implemented in some countries, given the national radiation protection. In our study, the ax‐pCR rate in MARI group was significantly lower than others. Whether the titanium‐encapsulated radioactive seeds would affect the uptake and metabolism of medicine in axillary nodes, or the phenomenon was due to the difference in initial tumor burden and neoadjuvant efficacy in populations, should be interpreted with caution. Compared to MARI, clips were cheaper and had better image visualization on either ultrasound, mammography, or computerized tomography. Commonly used marker clips in the research were made of metallic or specific bioabsorbable polymers (e.g., hydrogel). Preoperative imaging‐guided percutaneous wire localization and intraoperative ultrasonography markedly improved the IFR of clipped nodes. Disadvantages included rare allergic reactions and decreased ultrasound visibility of the hydrogel clips over time.^5^ Still, both clips and iodine seeds might fall out to the adjacent fat during arm movements and response to NST, if not be centrally placed in the cortex of nodes, and meanwhile at the risk of surgical complications containing seroma, hematoma, and affected margins.^42^ Additionally, long implantation time, up to 3 months to half a year, caused cystic changes and atypical granulomas within the marked nodes, making it difficult for pathologists to assess chemo response and even misdiagnosis them with metastatic cells.^34^, ^37^

The third method, suspended carbon particles, was a liquid black dye for labeling nodes at no risk of dislodgement and with a low potential for causing foreign body reactions.^43^ It showed unexpectedly excellent detection rates without any positioning support, with most studies reporting success rates as high as 100%.^43^, ^44^, ^45^, ^46^, ^47^ One study in the carbon group presented an extremely low IFR of 63.3%, probably due to the difference in physician operating techniques between participating institutions, suggesting learning curves should be considered when planning future researches.^48^ Another concern was that as the time interval between dye injection and surgery increased, the effect of dye diffusion along the lymphatic vessels to the adjacent adipose tissue and lymph nodes became non‐negligible. Reports from Spautz et al. also reflected that from the final surgical specimens, the colored nodes were more than initially labeled in nearly half of patients.^47^ An appropriate injection dose of 0.2–0.4 ml per node might reverse this dilemma while reducing the impact on searching blue‐stained SLN and the risk of severe postsurgical complications caused by removing excessive biopsied nodes.^43^

Small data on marking nodes with magnetic seeds and SAVI SCOUT (using infrared light and a microimpulse radar reflector) were reported.^49^, ^50^, ^51^ Their advantage was more pinpoint positioning, while the disadvantage was a poorer exploration of deep nodes due to the limitations of unique detectors. Radiofrequency identification (RFID) devices were likewise reported in TLNB, with only scarce evidence.^52^ Its distinct benefits were that tags could be distinguished and used to label different nodes in the same region.

A combination of TLNB and SLNB should be recommended. Siso et al. reported a case of axillary failure in that only one SLN was removed, and it overlapped with the sole TLN.^37^ Several studies supported a higher incidence of FN in SLNB + TLNB cohorts when one of the operations failed.^25^, ^36^, ^53^ Correspondingly, we came to the same conclusion that the combined group had a better FNR than the single group (5.1% vs. 6.3%), and the IFR (94.5% vs. 88.8%). The median number of SLNs was less than three in a majority of the combined group, suggesting that SLNB alone could not reliably predict residual axillary tumor in more than half of the patients.^22^, ^24^, ^43^, ^47^, ^53^, ^54^ Clearly, the advantage of TLNB for ensuring the number of biopsied nodes was realized, which gave SLNB more room for error and allowed more patients to achieve more than three biopsied nodes after neoadjuvant therapy.^11^, ^14^, ^55^

Micrometastasis and isolated tumor cells deposited in nodes after NST, which were presumed to mirror stronger tumor resistance to drugs, were likely missed by the regular frozen section.^4^ According to previous data, if micrometastasis and isolated tumor cells were presented in biopsied nodes after NST, 60% and 17% of cases might have additional ALNs metastases.^56^ Though Chun et al. reported that in 98 patients with no more than two positive SLNs after NST, replacing ALND to axillary radiotherapy without compromising survival outcomes, most scholars remained vigilant about micro residuals in post‐NST nodes and recommended omitting ALND only in ypN0 patients.^57^ IHC, cytokeratin staining, and intraoperative touch imprint cytology (ITPC) were more sensitive to identifying and rechecking the negative biopsied nodes, and routine application of these technologies might reduce the misdiagnosis of pathologists.^26^, ^37^

Interestingly, we found initial nodal status and the overlap rate of targeted and sentinel biopsied nodes as potential reflectors of the high‐qualified successful biopsy after NST. ACOSOG Z1071 reported that among patients with initial cN1 and ≥2 SLNs resection, FNR was 6.8% when clipped nodes were contained in SLNs, and up to 19.0% when they were not.^58^ The same results were reported by Cabioglu et al. that FNR decreased with an intraoperative finding of TLNs as SLNs (10.3% vs. 16.7%), especially in patients with initial cN1 (4.2% vs. 16.7%).^53^ On the contrary, patients with high axillary metastatic burden (more than 4 abnormal nodes on ultrasound before NST, ≥cN2) were less likely to have TLNs corresponding to SLNs.^37^, ^59^ In our study, the coincidence rate of TLNs and SLNs ranged from 35.7% to 87.5%, among whom groups with high overlap rates and high initial cN1 rates tended to achieve superior IFRs and FNRs.^24^, ^44^ We considered this could be explained from the aspect of anatomy. According to the axillary lymphatic drainage pathway, SLNs were the first station to be attacked when tumors started to spread.^1^, ^60^ While the location of the TLN directly reflected the infiltrating degree of the tumor cell in the axilla.^22^, ^24^, ^61^ That is, when the most typical metastatic nodes at aspiration were restricted to the first stop of tumor lymphatic reflux, the tumorous axillary invasion was still in its early stage, both of which mirrored a low tumor load in the ALNs. Different from the past, we found that subjects without abnormal axillary nodes on imaging after NST (ycN0) did not achieve improved detection and diagnostic efficacy.^23^, ^44^, ^53^ Integrating the limitations of noninvasive axillary imaging alone in determining therapeutic response, we believe utilizing them to identify initial cN1 patients before NST was more desirable.^62^

Given tumor heterogeneity, the response to NST and imaging manifestation varied considerably in different molecular subtypes, which should also be integrated into a potential decision algorithm. Studies showed high synchronicity of remission between the primary lesion and metastatic axillary nodes that 100% of initial cN0 and 89.6% of initial cN1 patients achieved ax‐pCR when they achieved breast pCR.^63^ The synchronous rate was more excellent in Her‐2‐amplified and triple‐negative subtypes, from a review of 30,821 subjects reported by Mayo Clinic.^64^

Furthermore, to answer whether additional axillary management could be safely exempted in patients with initial high axillary tumor load but achieving ax‐pCR on TAD after NST, a stepwise treatment strategy combining imaging and radiotherapy should be recommended. Morrow et al. reported that among 206 subjects with three or more negative SLNs and omitted ALND after NST, only one refused radiotherapy, and she was also the only subject who experienced nodal recurrence.^65^ Inspiringly, Koolen et al. proposed four or more abnormal nodes on pre‐NST PET/CT as a critical point for distinguishing initial axillary tumor load.^36^ Additional axillary radiotherapy was added for initial low‐load patients with residual tumors after NST and those who achieved ax‐pCR with initial high tumor load and was exempted only in initial low‐load patients with pathological negative TLNs after NST. The constructed algorithm ultimately resulted in 74% of patients avoiding ALND.

The study had some limitations. First, the sample size of TLNB group was still small, and since most trials had performed standard ALND after TLNB, long‐term follow‐ups on survival and quality of life after removing ALND to more minor de‐escalated treatments were lacking. The ongoing multicenter, randomized phase‐III TAXIS trial that assessing the antitumor security of tailored axillary surgery plus axillary radiotherapy compared to ALND, could offer greater completeness for scientific analysis in the future. Additionally, cause macrophages and lymphocytes after NST were likely to be confused with tumor residual on routine sectioning, we could not recheck the positive biopsied results and recognized the possible overtreated cases with negative ALNDs. Finally, surgical standards of TAD remained to be unified, such as the timing of marking, selection criteria when more than one suspicious lymph node presented, management after a failed biopsy.

In conclusion, TLNB is a novel de‐escalated surgical approach to distinguish initial N+ breast cancer that achieves axillary negative conversion after NST. It yields a low FNR of 5.5%, a high NPV of 93.4%, and a remarkable IFR of 93.5%, which has a broad prospect of clinical application. A combination of preoperative imaging, intraoperative TLNB with SLNB, and postoperative radiotherapy might affect the future treatment paradigm of primary breast cancer with nodal metastases.

## CONFLICT OF INTEREST

The authors declare that they have no known competing financial interests or personal relationships that could have appeared to influence the work reported in this paper.

## AUTHORS’ CONTRIBUTIONS

Yu‐xin Song carried out conceptualization, literature review, data collection, statistical analysis, writing—original draft, and writing—review and editing. Zheng Xu carried out literature review, data collection, and statistical analysis. Ming‐xing Liang carried out methodology and writing—review and editing. Zhen Liu verified the data and analytical methods. Jun‐chen Hou verified the data and analytical methods. Xiu Chen was involved in statistical analysis. Di Xu carried out supervision. Yin‐jiao Fei verified the data and analytical methods. Jin‐hai Tang was involved in conceptualization. All authors discussed the results and contributed to the final article.

## ETHICAL APPROVAL STATEMENT

No ethical approval is required since this study retrieved and synthesized data from already published studies.

## Supporting information


Appendix S1

## Data Availability

The data that support the findings of this study are available from the corresponding author upon reasonable request.
