# Supplementary material for: Diagnostic accuracy of de‐escalated surgical procedure in axilla for node‐positive breast cancer patients treated with neoadjuvant systemic therapy: A systematic review and meta‐analysis
Source: Cancer Med. 2022 May 3;11(22):4085–103. doi: 10.1002/cam4.4769 (PMC9678111; doi:10.1002/cam4.4769)

Supplementary 1. Searching strategies

PubMed search strategy

(((((Lymph Nodes[MeSH Terms]) OR (lymphatic metastasis[MeSH Terms]) OR (lymph node[Title/Abstract])) OR (axilla[MeSH Terms]) OR (axilla*[Title/Abstract])) AND ((((((((Neoadjuvant Therapy[MeSH Terms] ) OR (Neoadjuvant Therapies[Title/Abstract] OR Therapies, Neoadjuvant[Title/Abstract] OR Therapy, Neoadjuvant[Title/Abstract] OR Neoadjuvant Treatment[Title/Abstract] OR Neoadjuvant Treatments[Title/Abstract] OR Treatment, Neoadjuvant[Title/Abstract] OR Treatments, Neoadjuvant[Title/Abstract]))) ）) OR (chemotherapy[Title/Abstract])) OR (targeted therapy[Title/Abstract])) OR (endocrine therapy[Title/Abstract]))) AND (sensitiv*[Title/Abstract] OR sensitivity and specificity[MeSH Terms] OR (predictive[Title/Abstract] AND value*[Title/Abstract]) OR predictive value of tests[MeSH Terms] OR accuracy*[Title/Abstract])) AND ((Sentinel Lymph Node Biopsy[MeSH Terms]) OR (Lymph Node Biopsy, Sentinel) OR (Biopsy, Sentinel Lymph Node) OR (Tattoo[Title/Abstract]) OR (targeted axillary dissection[Title/Abstract]) OR (MARI[Title/Abstract]) OR (clip[Title/Abstract]))) AND (((breast neoplasm[MeSH Terms])) OR (((breast[Title/Abstract]) OR (mamma*[Title/Abstract])) AND (((((cancer[Title/Abstract]) OR (carcinoma[Title/Abstract])) OR (tumor[Title/Abstract])) OR (tumour[Title/Abstract])) OR (neoplasm[Title/Abstract]))))

EMBASE search strategy

(tatoo:ab,ti OR 'targeted axillary dissection':ab,ti OR mari:ab,ti OR 'sentinel lymph node biopsy'/exp) AND ('neoadjuvant therapy'/exp OR chemotherapy OR 'targeted therapy' OR 'endocrine therapy') AND ('breast tumor'/exp OR ((breast OR mamma) AND (tumor OR tumour OR cancer OR carcinoma))) AND ((axilla*:ti,ab OR lymph*:ti,ab) OR (node*:ti,ab OR metastasis:ti,ab)) AND (sensitiv*:ti,ab OR diagnostic accuracy:ti,ab OR diagnostic:ti,ab)

Cochrane search strategy

#1 MeSH descriptor: [Breast Neoplasms] explode all trees

#2 (breast): ti,ab,kw OR (mamma*):ti,ab,kw

#3 (cancer): ti,ab,kw OR (carcinoma):ti,ab,kw OR (tumor):ti,ab,kw OR (tumour):ti,ab,kw OR (neoplasm):ti,ab,kw

#4 #1 OR (#2 AND #3)

#5 ((targeted): ti,ab,kw AND (dissection):ti,ab,kw) OR (tatoo):ti,ab,kw OR (MARI):ti,ab,kw OR (clip):ti,ab,kw OR (mark):ti,ab,kw OR (iodine):ti,ab,kw

#6 MeSH descriptor: [Neoadjuvant Therapy] explode all trees

#7 (chemotherapy): ti,ab,kw OR (targeted therapy):ti,ab,kw OR (endocrine therapy):ti,ab,kw

#8 #6 OR #7

#9 (axilla*): ti,ab,kw OR (lymph):ti,ab,kw OR (node*):ti,ab,kw OR (metastasis):ti,ab,kw OR (positive):ti,ab,kw

#10 #9 AND #8 AND #5 AND #4

Figure S1. Forest plot of the ax-pCR rate of TAD, grouped by the proportion of Her-2 positive and triple-negative subtypes

0: studies with the proportion of Her-2 positive and triple-negative subtypes < 50%; 1: studies with the proportion of Her-2 positive and triple-negative subtypes > 50%.

Figure S2. (A) Forest plot of the positive predictive value of TAD; (B) Forest plot of the false positive value of TAD (at the circumstance that false positives were not adjusted to 0)


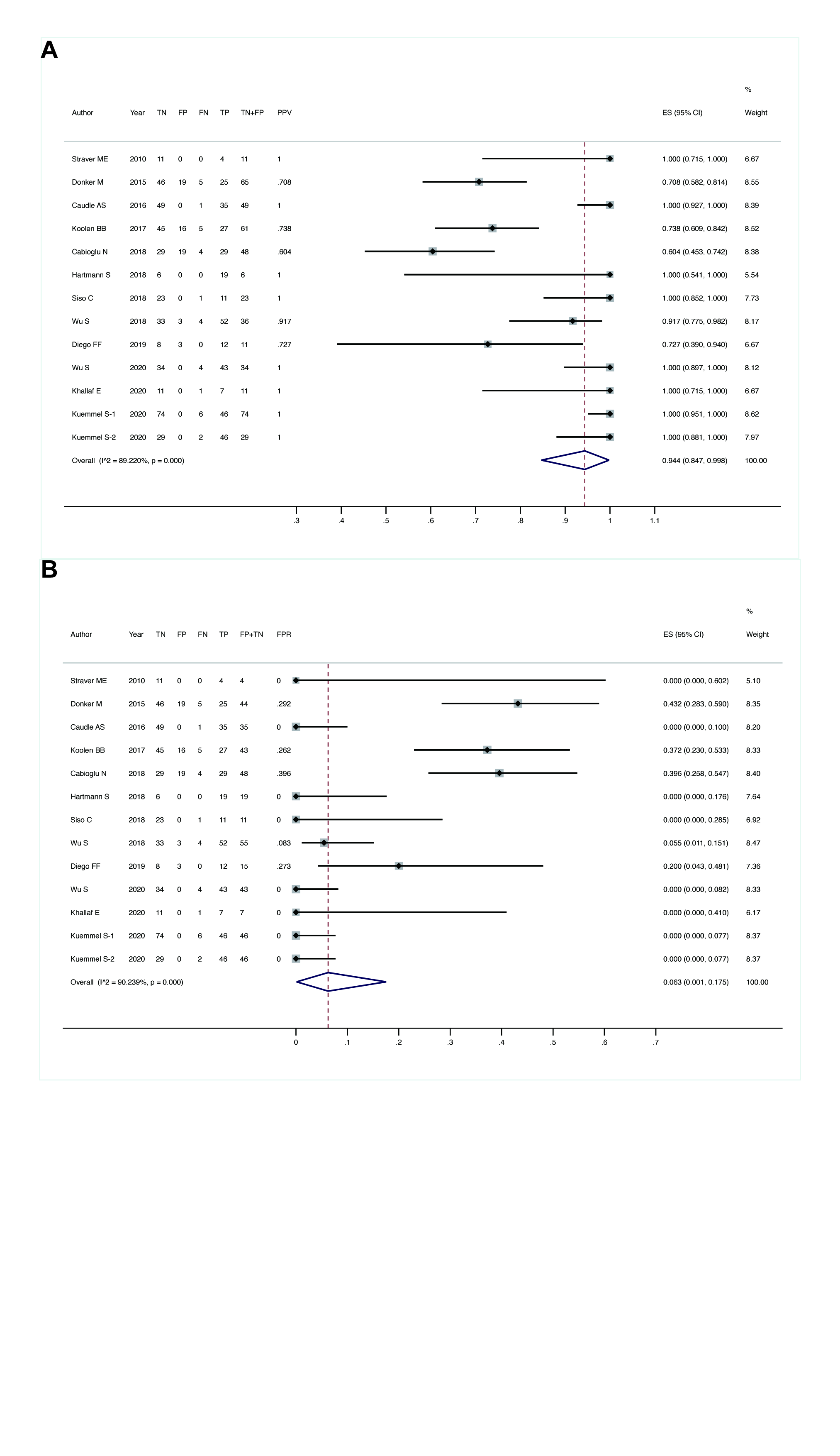

Supplement: Supplementary file 1 — Appendix S1 [file CAM4-11-4085-s001.docx]
